# Development of synchronous VHL syndrome tumors reveals contingencies and constraints to tumor evolution

**DOI:** 10.1186/s13059-014-0433-z

**Published:** 2014-08-27

**Authors:** Rosalie Fisher, Stuart Horswell, Andrew Rowan, Maximilian P Salm, Elza C de Bruin, Sakshi Gulati, Nicholas McGranahan, Mark Stares, Marco Gerlinger, Ignacio Varela, Andrew Crockford, Francesco Favero, Virginie Quidville, Fabrice André, Carolina Navas, Eva Grönroos, David Nicol, Steve Hazell, David Hrouda, Tim O’Brien, Nik Matthews, Ben Phillimore, Sharmin Begum, Adam Rabinowitz, Jennifer Biggs, Paul A Bates, Neil Q McDonald, Gordon Stamp, Bradley Spencer-Dene, James J Hsieh, Jianing Xu, Lisa Pickering, Martin Gore, James Larkin, Charles Swanton

**Affiliations:** Cancer Research UK London Research Institute, London, WC2A 3LY UK; Royal Marsden NHS Foundation Trust, London, SW3 6JJ UK; University College London Cancer Institute, London, WC1E 6DD UK; Centre for Mathematics & Physics in the Life Science & Experimental Biology (CoMPLEX), University College London, London, WC1E 6BT UK; Centre for Evolution and Cancer, Institute of Cancer Research, London, SW7 3RP UK; Instituto de Biomedicina y Biotecnología de Cantabria (CSIC-UC-Sodercan), Departamento de Biología Molecular, Universidad de Cantabria, Santander, 39011 Spain; Cancer System Biology, Center for Biological Sequence Analysis, Department of Systems Biology, Technical University of Denmark, Lyngby, DK-2800 Denmark; Institut Gustave Roussy, Villejuif, 94805 France; Imperial College Healthcare NHS Trust, London, W6 8RF UK; Guy’s and St Thomas’ NHS Foundation Trust, London, SE1 9RT UK; Department of Human Oncology and Pathogenesis Program, Memorial Sloan Kettering Cancer Center, New York, 10065 USA

## Abstract

**Background:**

Genomic analysis of multi-focal renal cell carcinomas from an individual with a germline *VHL* mutation offers a unique opportunity to study tumor evolution.

**Results:**

We perform whole exome sequencing on four clear cell renal cell carcinomas removed from both kidneys of a patient with a germline *VHL* mutation. We report that tumors arising in this context are clonally independent and harbour distinct secondary events exemplified by loss of chromosome 3p, despite an identical genetic background and tissue microenvironment. We propose that divergent mutational and copy number anomalies are contingent upon the nature of 3p loss of heterozygosity occurring early in tumorigenesis. However, despite distinct 3p events, genomic, proteomic and immunohistochemical analyses reveal evidence for convergence upon the PI3K-AKT-mTOR signaling pathway. Four germline tumors in this young patient, and in a second, older patient with VHL syndrome demonstrate minimal intra-tumor heterogeneity and mutational burden, and evaluable tumors appear to follow a linear evolutionary route, compared to tumors from patients with sporadic clear cell renal cell carcinoma.

**Conclusions:**

In tumors developing from a germline *VHL* mutation, the evolutionary principles of contingency and convergence in tumor development are complementary. In this small set of patients with early stage VHL-associated tumors, there is reduced mutation burden and limited evidence of intra-tumor heterogeneity.

**Electronic supplementary material:**

The online version of this article (doi:10.1186/s13059-014-0433-z) contains supplementary material, which is available to authorized users.

## Background

Von Hippel-Lindau (VHL) disease is an autosomal dominant hereditary cancer syndrome attributed to germline mutation of the *VHL* gene, located on chromosome 3p25 [1,2]. The clinical phenotype is characterized by the development of vascular tumors, including hemangioblastomas of the central nervous system and retina, clear cell renal cell carcinomas (ccRCCs) and pheochromocytomas. In the kidney, loss of the remaining wild-type *VHL* allele, consistent with a ‘two-hit’ model of tumor suppressor inactivation [3], results in multiple pre-neoplastic cysts and ccRCC tumors. In sporadic ccRCC, somatic mutation or hypermethylation of *VHL* occurs in the majority [4–6] and 3p loss of heterozygosity (LOH) in over 90% of cases [6–8]. Furthermore, recent work from our group has demonstrated that 3p LOH and *VHL* mutation or methylation are early truncal (ubiquitous) events in 10 of 10 cases of sporadic ccRCC subjected to multi-region sequencing (M-seq) [9]. Biallelic inactivation of the tumor suppressor gene *VHL* is therefore established as an early event in both germline mutant *VHL*-associated and sporadic ccRCC [6,8–11].

Next-generation DNA sequencing has identified an additional set of tumor suppressor genes that are recurrently mutated in sporadic ccRCC [6,7,11–14]. Several of these (*PBRM1*, *SETD2* and *BAP1*) are also located on chromosome 3p, emphasizing the importance of this locus for ccRCC tumorigenesis. A second striking feature of this catalogue of somatic events is that tumor suppressor genes involved in chromatin modification, including *PBRM1*, *SETD2*, *KDM6A* and *JARID1C* [15], and components of the phosphoinositide 3 kinase (PI3K)-AKT-mammalian target of rapamyocin (mTOR) pathway (for example, *PTEN*, *PIK3CA*, *AKT*, *TSC1/2* and *MTOR*), feature prominently. Whole exome sequencing of multiple regions of primary ccRCC has shown that anomalies in these genes may occur subclonally, separated spatially within the same primary tumor. Despite profound intra-tumor heterogeneity, parallel evolution of subclones occurs with convergence of distinct mutations occurring in the same gene, signal transduction pathway or protein complex [9,11], within a branched evolutionary model of cancer development [16]. It is evident that the branching pattern of mutational events in ccRCC (and other cancers) is highly complex, and varies significantly between patients with the same histopathological tumor type [9]. Importantly, cancer cells do not evolve in isolation but in the context of the tissue eco-system. Selection can therefore be influenced by multiple environmental factors (reviewed in [17,18]).

The genomic analysis of synchronous or metachronous tumors occurring in the same organ in one individual offers a rare opportunity to relate evolutionary principles to tumorigenesis. Cancers that develop from an identical germline abnormality, arising in the same organ, and that are subject to the same environmental constraints are analogous to a hypothetical experiment proposed by the paleontologist and evolutionary biologist Stephen Jay Gould. Gould summarized his view of the history of life and multi-cellular organism evolution by challenging that if the ‘tape of life’ were to be re-wound to the same starting point and re-played under the same conditions, the evolutionary trajectory would follow a markedly different course to the one actually taken [19]. This does not imply that evolution is random, but rather that the final result is causally dependent upon the sequence of antecedent steps, a notion Gould termed ‘historical contingency’. Convergence, or the idea that evolutionary constraint results in a limited set of outcomes, may be cited as an opposing theory to contingency [20]. Here we use whole exome sequence data from a patient with a germline *VHL* mutation and synchronous ccRCC tumors to add experimental evidence for contingency, but also find that contingency and convergence are not mutually exclusive but rather complementary. We propose that in this patient, the development of independent primary kidney cancers in the context of a germline *VHL* mutation occurs by a different sequence of genomic abnormalities in each tumor, contingent upon the nature of a chromosome 3p LOH event occurring early in tumorigenesis. Ultimately, however, the tumor trajectories converge to cause PI3K-AKT-mTOR pathway activation, via somatic mutation of components of this pathway and alternative mechanisms. Surprisingly, in contrast to 10 sporadic ccRCCs from older patients, the three assessable tumors follow linear, rather than branched, evolutionary routes and have few somatic mutations. We suggest this is due to the consequence of the young age and early tumor stage at diagnosis, following cyst surveillance due to the germline VHL syndrome, allowing limited time for acquisition of passenger mutations and subclonal expansion, contributing to spatial heterogeneity.

## Results

### Clinical case report

**Figure 1:**
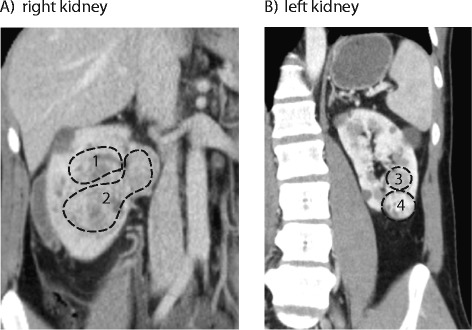
**Multi-focal renal cell carcinoma tumors in a patient with VHL disease.** Coronal sections from computed tomography scans show the spatial orientation of the tumors within the kidneys. **(A)** A macroscopically single tumor in the right kidney, but defined by exome sequencing as two tumors (one and two). **(B)** Two separate tumors in the left kidney, tumors three and four. Cysts typical of VHL disease are also present in both kidneys.

A 32-year-old male presented with hematuria. Ultrasound demonstrated multiple renal cysts and the initial diagnosis was polycystic kidney disease. However, the cysts enlarged during a surveillance period of one year, leading to computed tomography imaging demonstrating a locally advanced right renal tumor, and two tumors in the left kidney (Figure 1). Further investigation revealed capillary retinal angiomas and hemangiomas of the brain and spine typical of VHL disease, and he was diagnosed with a *de novo*, germline nonsense mutation in exon 3 of the *VHL* gene. At the age of 33, the patient underwent a radical right nephrectomy; histopathological examination confirmed a single stage 3, Fuhrman grade 3 ccRCC (pT3aN0M0). Six months later, there was progression in the left kidney and he was treated with partial left nephrectomy to remove both tumors. Both were stage 1 ccRCCs with a maximum Fuhrman grade of 2 (pT1aN0M0). At the time of this report, the patient remains in complete remission.

### Whole exome sequencing defines four tumors of independent clonal origin

We harvested multiple regions from each tumor on the right and left sides, as well as normal kidney tissue, attempting to sample the spatial extent and morphological heterogeneity of each renal cell carcinoma. Whole exome sequencing (WES) to a median depth of 87× was performed on seven tumor regions from the right side (representing one morphological tumor with a heterogenous macroscopic appearance), and on nine tumor regions from the left side (representing two, macroscopically distinct but homogenous tumors). We used the CaVEMan algorithm to detect non-synonymous somatic mutations [12], referenced against germline DNA extracted from peripheral blood and sequenced to a depth of 94×. Small insertions and deletions (indels) were identified using Pindel, described previously [9]. All candidate mutations were subjected to orthogonal validation by ultra-deep amplicon sequencing, to an average depth of 254×. A non-synonymous mutation or indel was verified if the variant allele occurred in at least 1% of reads.

**Figure 2:**
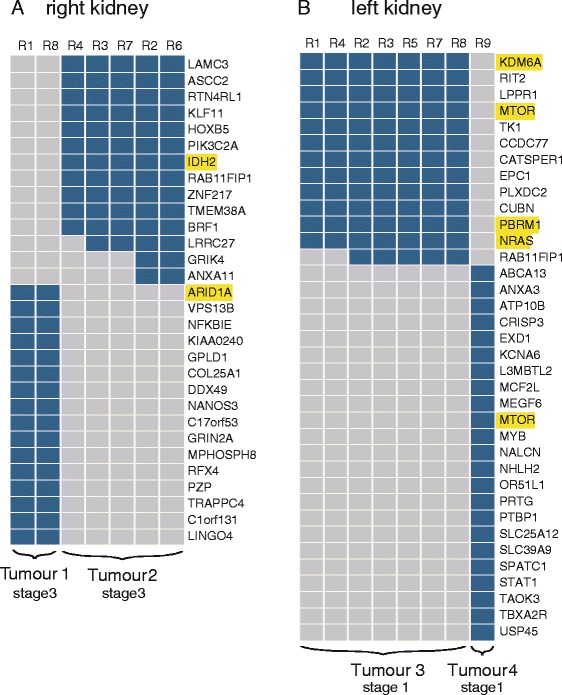
**Intra-tumor heterogeneity in four germline tumors. (A,B)** Heatmaps show the regional distribution of all non-silent mutations; presence (blue) or absence (grey) of each mutation is indicated for every tumor region in tumors from the right **(A)** and left kidneys **(B)**. Whole exome sequencing defines four distinct tumors. In each, tumor evolution is linear rather than branched.

A nonsense mutation was confirmed at codon 120, exon 3 (R120*) in the *VHL* gene, present in the blood, normal kidney tissue and all tumor regions. All tumor regions also displayed 3p LOH, which we have previously found to be the only common early event in sporadic clear cell renal tumorigenesis together with *VHL* mutation or methylation [9]. In addition, WES identified 97 non-synonymous mutations, including 11 indels, present in at least one tumor region. Validation was attempted for all 97 putative mutations; 16 variants did not validate (validation rate 83.5%). Validation data for a further 15 variants were considered inconclusive, either because an amplicon could not be generated or there was insufficient read coverage (<50×) and these were excluded from further analysis. The spatial distribution of validated somatic mutations clearly defined four, rather than three, tumors of distinct clonal origin (two on each side) - hereafter referred to as tumors one and two on the right side and three and four on the left side (Figure 2; Additional file 1: Table S1).

### Parallel tumor evolution and distinct 3p LOH events in each of the four ccRCC tumors

**Figure 3:**
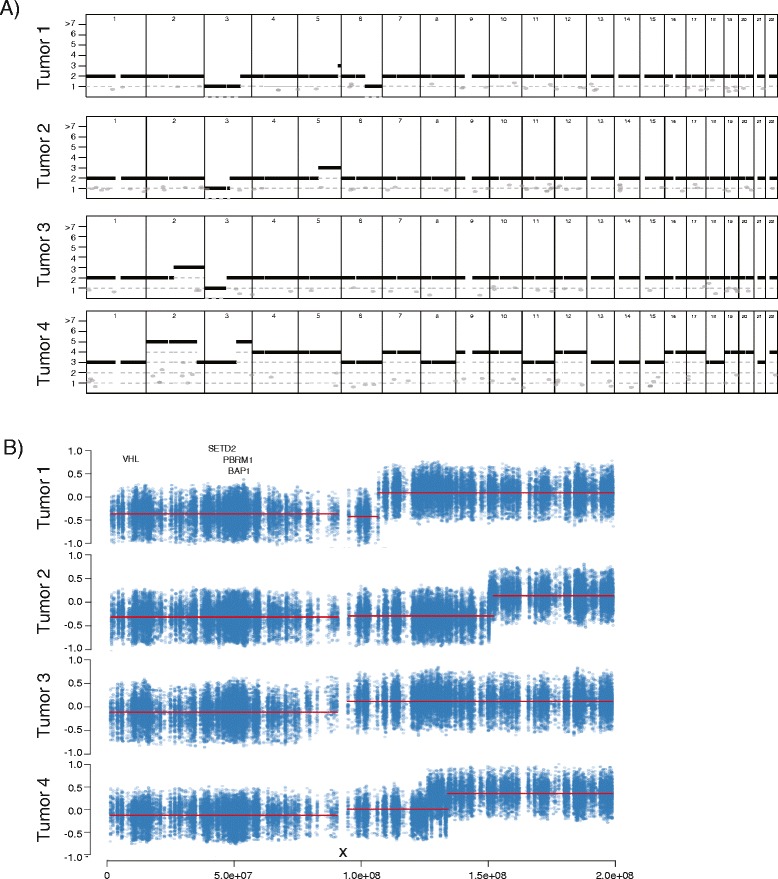
**Copy number analysis in four germline tumors. (A)** Copy number profiles of representative samples from each of the four tumors, with integer copy number on the x-axis. Loss of chromosome 3p is ubiquitous. **(B)** The chromosome 3p breakpoint locations for the four tumors. Each y-axis is logR, and the x-axis represents position along chromosome 3 in hg19. An ‘X’ near the x-axis marks the approximate position of the centromere.

We determined the tumor ploidy of each region by flow cytometry and implemented ABSOLUTE [21] to estimate copy number aberrations (CNAs) in each of the tumor regions. We observed ubiquitous 3p LOH in all tumor biopsies from this patient, consistent with this being an early founder event common to all clear cell carcinomas [9] (Figure 3A; Additional file 2).

However, we identified four distinct CNA profiles defined by differing breakpoint locations in chromosome 3p and additional CNAs; these four profiles correspond to the four distinct tumors delineated by somatic mutation analysis (Figure 3B). For tumor one from the right kidney, the chromosome 3p breakpoint mapped to *CLRN1.* However, in the ipsilateral tumor two, the breakpoint fell in the vicinity of *ALCAM*. Additionally, tumors one and two differ by their loss of 6q and gain of 5q, respectively. On the left side, tumor three demonstrated loss of 3p with a breakpoint in the centromere as well as gain of 2q. Comparatively, the copy number profile in tumor four was complex and was suggestive of genome doubling. This was confirmed by flow cytometry assessment of ploidy, demonstrating that tumor four was near-tetraploid with a DNA index of 1.82 (Additional file 3). The 3p breakpoint in tumor four mapped to the region of *BFSP2*, and there were a number of additional events, including gains of chromosomes 2, 5, 7, 9, 10, 12, 16 and 17.

### Phylogenetic tree analysis reveals distinct, linear evolutionary trajectories of hereditary VHL ccRCCs

We defined a driver event as a somatic mutation in a gene or CNA previously reported to occur in ccRCC [6,7,9,11–13,22]. Each non-synonymous mutation was assigned a category of 1 to 3 according to published criteria (category 1, high confidence driver mutation; category 2, probable driver mutation; category 3, mutation of unknown significance) [9]. Apart from 3p LOH, somatic driver events differed between the four tumors delineated by WES (Figure 2). For example, in the right kidney, somatic mutations were identified in *ARID1A* in tumor one (category 2), and in *IDH2* in tumor two (category 3). In tumors three and four, there was a distinct somatic mutation in *MTOR (*categories 2 and 3, respectively) in each tumor, and *PBRM1* and *KDM6A* and *NRAS* mutations (all category 3) in tumor three. Subsequent to *VHL* mutation and loss of 3p, no single copy number or mutational event was common to all four tumors, suggesting contingency and the unpredictable nature of tumor evolution subsequent to a similar germline event.

Intriguingly, in contrast to sporadic clear cell carcinomas, intra-tumor heterogeneity (ITH) in germline-associated tumors in this patient was not profound and linear, rather than branched, evolution appears to occur in at least two of the four tumors (tumors two and three) in this young patient subjected to renal surveillance. In the stage 1 tumor three from the left kidney, 93% (14/15) of somatic mutations and copy number events were ubiquitous (present in all seven tumor regions). The remaining somatic mutation in *RAB11FIP1* was shared by five of seven tumor regions. In the stage 3 tumor two on the right side, 81% (13/16) of mutations and copy number events were ubiquitous, and 19% (3/16) heterogenous. However, among the three mutations that were not present in the phylogenetic trunk there was no branching between spatially separated tumor biopsies, consistent with a linear model of tumor evolution.

We calculated an index of branch to truncal mutations (ITB) for assessable tumors by dividing the mean number of non-ubiquitous mutations by the total number of ubiquitous mutations per tumor biopsy, and compared the indices for this patient with those of sporadic ccRCC tumors analyzed by M-seq previously [9] (Table 1). In this young patient with germline *VHL* mutant tumors, the ITB indices for both the stage 1 and stage 3 tumors fall within the low end of the range in this small cohort.

**Table 1:** **Branch:trunk indices (ITB) from M-seq for germline** ***VHL*** **mutant and sporadic ccRCC tumors**

**Tumor code**	**Age of patient (years)**	**Germline or sporadic** ***VHL*** **mutation/methylation**	**Tumor stage**	**ITB**
Tumor 3 (left)	33	Germline	I	0.06
EV006	65	Sporadic	IV	0.07
EV003	64	Sporadic	IV	0.17
Tumor 2 (right)	33	Germline	III	0.19
EV007	59	Sporadic	IV	0.56
RMH008	64	Sporadic	III	0.64
EV002	59	Sporadic	IV	0.67
EV005	79	Sporadic	IV	0.75
EV001	75	Sporadic	IV	0.84
RMH002	63	Sporadic	IV	0.93
RMH004	61	Sporadic	IV	1.81
RK26	47	Sporadic	II	2.35

### Reduced mutational load in germline VHL tumors

We defined the mutational load of a tumor as the number of non-synonymous somatic mutations per tumor biopsy; in this patient, the median mutational load in the stage 1 and stage 3 tumors was 13 (range 13 to 23). We hypothesized that the few somatic mutations observed in this patient, compared with a large cohort reported by The Cancer Genome Atlas (TCGA) [6], was a consequence of lower tumor stage and/or the young age at presentation. To further investigate this, we performed WES, to a median depth of 86x, on two samples of a homogeneous stage 1 ccRCC removed from a male patient aged 67 years with VHL syndrome (Additional file 4). Average life expectancy in VHL syndrome is only 49 years with renal cell carcinoma the leading cause of mortality [23]; therefore, ccRCC tumors from patients with a comparable age to the median age of diagnosis in sporadic ccRCC (63 years) are only rarely available. We confirmed a germline VHL mutation, as well as loss of 3p and gain of 5q in both tumor regions. Surprisingly, the mutation burden in this older patient was also low; we validated 26 non-synonymous somatic mutations of 33 candidate mutations (validation rate 78.8%; Additional file 5). Of these 26 mutations, 25 (96%) were shared by both tumor regions. We could not identify a known ccRCC driver gene in the tumor biopsies from this second patient (Additional file 1: Table S2). Furthermore, there was a striking absence of somatic mutations in chromosome 3p genes in tumors from both VHL syndrome patients. This is in clear contrast to the somatic mutation spectrum observed in the cohort of sporadic ccRCC patients with locally advanced and/or metastatic tumors, all of which demonstrated branched evolution and harbored multiple somatic mutations in genes located on chromosome 3 [9,11]. It is noteworthy that these somatic events almost always occurred in the branches, and were therefore subclonal. For example, all the *SETD2* and *BAP1* somatic mutations and the majority of *PBRM1* mutations were found in the branches of the tumor phylogenetic trees. In contrast, the tumors from our syndromic VHL patients reported in this manuscript do not demonstrate branched evolution. We speculate that the absence of branched evolution and subclonal diversification is the reason we fail to see second hits in *SETD2*, *BAP1* and *PBRM1* in our study. Alternatively, the different patterns of driver mutations observed between syndromic and sporadic ccRCC may relate to renal surveillance and the removal of early versus late stage tumors, respectively, and conceivably, mutations in one or more of these genes may themselves drive branched evolution and tumor progression.

### Convergence upon the PI3K-AKT-mTOR pathway

**Figure 4:**
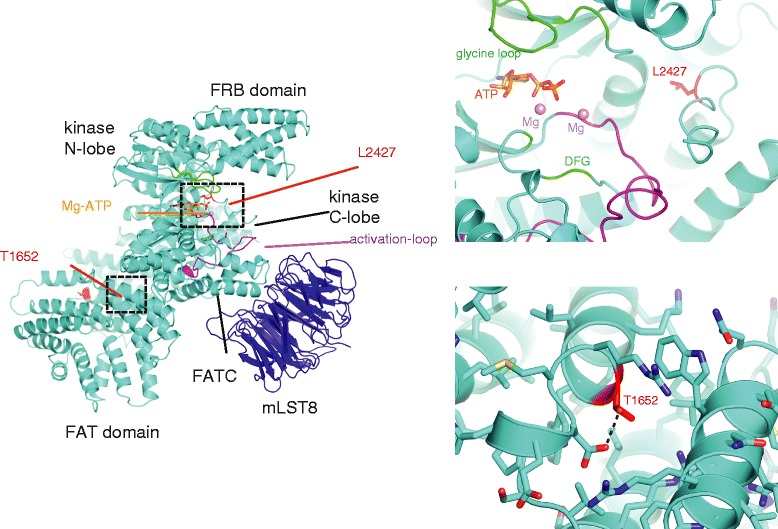
**Two distinct activating mutations in mTOR.** Left: a schematic of the mTOR protein structure (cyan) in complex with mLST8 (dark blue) (PDB code 4JSP). Key residues and structural features are highlighted close to mTOR mutations relevant to the current study. The FRB and FAT domains are also labeled together with the FATC sub-domain. Right: a close-up of regions in proximity to the mTOR mutation site L2427 abutting the nucleotide-binding cleft and activation loop (upper right panel). Lower right panel shows the environment surrounding residue T1652 in the FAT domain.

The two tumors in the left kidney harbored distinct point mutations in the *MTOR* gene (tumor three, p.L2427P; tumor four, p.T1652A). While only the first of these occurred in an identical amino acid encoded by a described alteration in *MTOR* [24–26], we predicted both to be hyper-activating based on their locations in the kinase and FAT (FRAP-ATM-TRAAP) domains of the mTOR protein, respectively, according to published studies [24–29] (Figure 4). However, after transfection of HEK-293 T cells with complementary DNA plasmids encoding wild-type or mutant (L2427P and T1652A) mTOR, only the L2427P mutant induced phosphorylation of S6K (Additional file 6). Phosphorylation of the other mTOR substrate, 4E-BP1, was similar between the wild-type, kinase domain and FAT domain mutant cells. These results confirm that the kinase domain mutation L2427P activates mTORC1 signaling, but suggest that the second mutation in the FAT domain, T1652A, does not. Indeed, T1652A is not reported elsewhere as a recurrent or functionally significant mutation; however, highly activating FAT domain mutations have been reported with experimental evidence suggesting that these may effect mTOR pathway activation by reducing binding between Deptor and mTOR [26].

**Figure 5:**
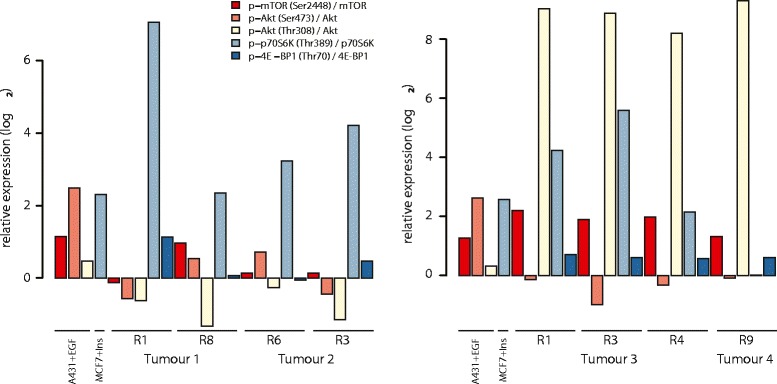
**Convergence upon the PI3K-AKT-mTOR pathway.** Graphs showing the ratio of phosphorylated to total protein for intra-cellular kinases of the PI3-AKT-mTOR pathway. For tumors one, two and three, more than one region from each tumor was analyzed.

We used a multi-array and Meso-Scale Discovery (MSD) technology to detect and quantify the levels of phosphorylated and non-phosphorylated intracellular kinase proteins of the PI3K-AKT-mTOR pathway. In all tumor regions, there was evidence of either S6K or 4EPB1 phosphorylation, consistent with pathway activation, relative to normal kidney from each side (Figure 5). In particular, S6K was phosphorylated in the tumor regions harboring the L2427P *MTOR* mutation. Additionally, immunohistochemical analysis of the mTOR substrates S6K and 4E-BP1 in all tumor regions revealed enhanced staining of either or both substrates relative to normal kidney in a pattern consistent with the proteomic data (Additional file 1: Table S3; Additional file 7). This is in contrast to absent staining of S6K and 4E-BP1 in *MTOR* wild-type tumor regions in a ccRCC previously reported [11]. We conclude that all tumors displayed functional convergence of mTOR pathway activation, despite distinct evolutionary somatic routes. Tumor four (harboring the FAT domain mutation) demonstrated markedly elevated phosphorylation of AKT, to a similar extent as induced in tumor three (harboring the kinase domain mutation). However, in tumor four there was increased phosphorylation of 4E-BP1 but not of S6K. Absent S6K phosphorylation would appear to be consistent with the lack of an activating mutation in mTOR in this region described earlier. There is evidence indicating that 4E-BP1 is regulated independently of S6K and it remains unclear whether the effect of AKT signaling on 4E-BP1 is wholly integrated by mTOR, or alternative kinases [30,31].

Similarly, in both tumors from the right kidney, without mutations in *MTOR*, there was increased phosphorylation of both S6K and 4E-BP1 substrates. However, phosphorylation of AKT or mTOR proteins relative to normal kidney tissue did not occur in tumors one and two. We propose that pathway activation in these tumors occurred by alternative mechanisms to *MTOR* mutation or AKT activation; mutation in *ARID1A* [32] or amplification of target genes on chromosome 5q, such as *GNB2L1* and *SQSTM1* [33–35], are both plausible explanations. Tumor two harbored a mutation in *PIK3C2A*, encoding the class II PI3K-C2α enzymes; the role of this enzyme in cell signaling and cell survival has been debated [36], but the most recent evidence indicates that it does not activate AKT [37]. In addition, mutations in *PIK3C2A* reported in cancer samples are distributed evenly across the protein and do not recurrently affect specific residues, suggesting these are passenger events [22].

Mutations in genes encoding components of the PI3K pathway and in *ARID1A* are reported at low frequencies in ccRCC (up to 6% and 3%, respectively) [6]. However, within this cohort, mutations in the PI3K pathway genes *EGFR*, *PTEN*, *PIK3CA*, *AKT*, *TSC1/2*, *RHEB*, and *MTOR* co-occur less frequently than would be expected by chance, as reported previously [6]. Further analysis reveals that mutations in *ARID1A*, *MTOR*, *TSC1* and *TSC2* show a similarly strong tendency towards mutual exclusivity (Additional file 8) and provides additional support for the role of *ARID1A* mutations in convergent activation of the PI3K pathway.

In summary, the proteomic and immunohistochemistry data illustrate clear convergence upon the PI3K-AKT-mTOR pathway, with increased signaling of the downstream substrates S6K and 4E-BP1, consistent with pathway activation in all tumors relative to normal kidney tissue. The mechanisms by which pathway activation occurred differed between tumors from the left and right kidneys and are likely to have been determined by distinct mutational or copy number events in each. In tumors without mutations in genes known to impact upon PI3K pathway signaling, we propose that alternative and as yet unknown mechanisms cause pathway activation based on substantial evidence for the integration of multiple regulatory inputs [31] and complex layers of control of the TORC1 complex, including the influence of the FAT domain mutation in the *MTOR* gene.

## Discussion

We report novel whole-exome sequencing data derived from metachronous, bilateral ccRCC tumors in a patient with VHL disease. Multi-focal tumors developing from the same germline, and in the same organ, are of independent clonal origin in this patient, despite identical histopathological characterization. Furthermore, genomic analysis reveals the presence of two distinct tumors in the right kidney, from what appeared macro- and microscopically to be a single ccRCC tumor. Our findings are consistent with an earlier report of copy number variation in 36 germline *VHL* mutant primary ccRCC tumors; within each of 12 patients, metachronous tumors displayed different copy number profiles, to the extent that tumors from the same patient were as dissimilar to each other as they were to tumors from other patients with VHL disease [38].

This clinical situation allows a rare insight into tumor evolution from the VHL mutant germline state in ccRCC, where the initiating event is the same across multiple tumors within the same organ and similar microenvironments; the combination of somatic mutation and copy number data defines a unique series of abnormalities for each of the four tumors. It is evident that, from a common starting point, there are multiple, currently unpredictable, routes to tumor development. Mutation of *VHL* was assumed to be the first hit, because of its presence in the germline in blood and normal kidney tissue. Based on previous data establishing that chromosome 3p loss is one of the founder events in ccRCC [8,9], and the ubiquitous nature of this event within the four tumors subjected to M-seq in this study, 3p LOH is likely to be the second hit. However, copy number data indicate that even amongst tumors arising from the same germline and in an identical environment, each of the four tumors harbored a distinct secondary 3p LOH event. Conceivably, the consequence of a different second event in the four tumors is to drive each through divergent trajectories; thus, subsequent events might be contingent upon the nature of the 3p event in these tumors. The notion that the final result of evolution is determined by antecedent steps is consistent with Gould’s theory of historical contingency. Of course, it is possible that an alternative driver event, such as 5q gain or somatic mutation, was the contingent step; however, on the basis that the 3p LOH is the only other ubiquitous event observed in sporadic cases and is ubiquitous in all tumors studied here, this is also likely to be one of the earliest events in VHL syndrome clear cell carcinoma.

Gould used the re-interpretation of the fossils of the Burgess Shale to illustrate an alternative view of life’s history, and offered the term contingency to encompass the idea of evolution as a series of unpredictable and unrepeatable events, but a system in which the final outcome is causally dependent on the occurrence of each antecedent step [19]. Gould referred to the concept of historical constraints within a lineage that limit the ability of natural selection to modify the future lineage; this notion of internal selective factors is cited by others in the field of developmental biology [39,40]. Our results support these statements by showing that histologically similar tumors derived from germline *VHL* mutation share 3p LOH as the second event but are then distinguished by their subsequent somatic genomic events. However, there may be constraints to their evolution, exemplified by the activation of the mTOR pathway in all tumor regions.

Naturally occurring or experimental evidence to support Gould’s claim of historical contingency is limited [41,42]. The molecular analysis of acute lymphoblastic leukemia in monozygotic twins provides support for his ideas [43–45]. Two separate twin studies now show that while the initiating event of a chromosomal fusion (even if not inherited) is the same, subsequent genomic aberrations differ substantially between cases of concordant and discordant acute lymphoblastic leukemia within monozygotic twin pairs, and this may have a profound bearing on clinical outcome. A second example from cancer medicine is in myelodysplastic syndromes, in which early driver mutations in different genes have been shown to direct disease evolution down ‘preferred’ trajectories, resulting in distinct clinical phenotypes [46]. In a simply designed long-term evolution experiment using 12 identical populations of *Escherichia coli* established in 1988, Lenski and colleagues managed to re-play the ‘evolutionary tape’ and thus provide unique evidence for historical contingency [47,48]. Further evidence for the evolution of specific protein functions being contingent upon permissive, ancestral mutations has been reported recently [49]. Results from our observational data support similar themes illustrated by these experiments: the development of a particular phenotype from an identical cell population and environment occurs via diverse, multi-step evolutionary pathways.

Conway Morris argued that evolution is constrained to such a degree that it converges upon a restricted range of options [20]. In this case, despite parallel evolution of the four individual tumors, there is indeed evidence for convergence upon the PI3K-AKT-mTOR pathway with two independent *MTOR* mutations in tumors from the left kidney and proteomic evidence for pathway activation in all tumor regions. Our data add to a compelling body of evidence for convergence upon this pathway, both within and between individual patients. A recent analysis of 106 ccRCC cases reported that the *MTOR* gene was mutated in nearly 6% of cases, but that a quarter of cases harbored mutations in genes encoding signaling molecules in the PI3K-AKT-mTOR cascade, including PTEN, PIK3CA, TSC1 and TSC2 [7]. Furthermore, the proportion of pathway mutations rises to 60% when calculated on a per patient, rather than per biopsy, basis [9].

Functional convergence on the mTOR pathway was evidenced by phosphorylation of downstream substrates S6K or 4E-BP1 in all four tumors relative to normal kidney, despite divergent somatic copy number and mutational events. *In vitro* experiments showed that the mutation in the FAT domain of mTOR was not activating; the functional significance of this mutation therefore remains unclear but theoretically it could influence mTOR-substrate interactions. It is likely that similar microenvironmental selection pressures across all four tumors contribute significantly to convergent signaling activity witnessed in this study. The identification of an *ARID1A* mutation in tumor one in the contralateral kidney to those tumors harboring *MTOR* mutations may further illustrate convergence upon the PI3K-AKT-mTOR pathway. Co-occurrence of mutations of this gene and those of the PI3K pathway are reported to occur in endometrial and head and neck cancers, and *ARID1A* mutations may impact upon PI3K pathway activity [32,50]. Although the mechanism is unknown, phosphorylation of PI3K downstream targets, including PDK1, AKT, TSC2 and S6K, was significantly upregulated in the presence of *ARID1A* mutations, and knockdown of wild-type *ARID1A* in three endometrial cancer cell lines resulted in significantly elevated phosphorylation of AKT [32]. Pathway activation in tumor two could have been the result of gain of chromosome 5q, which has been identified as a driver in ccRCC, occurring in 67% of samples [6]. Importantly, gain of amplicon 5q.35.3 results in alteration of two genes, *GNB2L1* and *SQSTM1*, whose overexpression is associated with activation of the PI3K pathway [6,33–35]. It is noteworthy that mutations in *MTOR* and in *GNB2L1* and *SQSTM1*, were shown to be mutually exclusive in ccRCC [6], consistent with pathway convergence as demonstrated by tumors one/two and three/four in this patient. Furthermore, we observed a strong tendency towards mutual exclusivity between mutations in *ARID1A* and PI3K pathway genes (*MTOR* and *TSC1/2*) [6]. In summary, we suggest that dysregulation of the PI3K pathway in this patient was achieved in all tumors but mediated by diverse mutational or copy number events.

In two patients with ccRCC tumors arising from germline VHL mutation, ITH was minimal despite extensive sampling in both a morphologically heterogeneous stage 3 tumor and a morphologically homogeneous stage 1 tumor. Within one of these patients, two evaluable tumors appeared to follow a linear, rather than branched evolutionary path. Furthermore, there was a noticeable absence of mutations in driver ccRCC genes, or second hits, in genes such as *SETD2*, *PBRM1* and *BAP1* in both patients. These patterns, distinct from those observed in tumors from patients with sporadic ccRCC, might be a direct consequence of a critical mutational event in the germline. Alternatively, early stage, non-metastatic disease may harbor less genetic ITH than advanced later stage tumors, although our recent cohort of sporadic ccRCCs included one stage 2 and one stage 3 tumor and both demonstrated ITH and branched evolution; in particular, the high ITB index for the stage 2 tumor testifies to this [9]. Further assessment of spatial ITH in early stage, node-negative, sporadic and germline *VHL* mutant tumors will be required to formally test these hypotheses.

## Conclusions

In germline *VHL* mutant ccRCC, historical contingency is illustrated by a different 3p LOH event in each of four clonally distinct tumors together with distinct driver events in each of the four tumors. Despite independent evolutionary trajectories, structural genomic and somatic events, selected within a similar microenvironment, converge to cause activation of the PI3K-AKT-mTOR pathway. Through the analysis of spatially and temporally separate ccRCC tumors from a young patient with germline *VHL* mutation, these data highlight the complementary properties of contingency and convergence during tumor evolution within an identical genetic background and tissue microenvironment.

## Materials and methods

### Patient samples

The patients consented to provide blood and tissue samples and clinical information, in a study approved by the National Research Ethics Committee London (Fulham, reference number 11/LO/1996) and the study was conducted in accordance with the principles of the Declaration of Helsinki. Both patients provided written consent to publication of the study results. At the time of nephrectomy surgery, samples of tumor tissue and normal kidney were collected according to strict protocols published previously [11]. For the first patient, from the right nephrectomy specimen, approximately 1 cm of tumor was taken from seven distinct regions, representing the spatial extent of the tumor and morphological heterogeneity. From the left partial nephrectomy specimen, two separate tumors were dissected from the kidney and provided whole. The first tumor was bisected and one half divided into eight regions. The second tumor was amorphous due to significant disruption during the surgical process and was processed as one sample. For the second patient, the largest tumor was bisected. Each tumor region was dissected using a fresh scalpel to avoid DNA contamination between samples, and all samples were snap-frozen within 60 minutes of ischemia. From each region, a matched sample was taken for histopathological assessment.

### Whole exome sequencing

DNA was extracted from blood and tissue samples using the Qiagen DNeasy kit and the manufacturer’s protocol (Manchester, United Kingdom The Beijing Genomics Institute performed initial quality control assessments, and then exome capture using the Agilent Human All Exome V4 kit (Cheshire, United Kingdom). Samples were paired-end multiplex sequenced on the Illumina HiSeq platform to a median target depth of >100×. Tumors from the second patient were sequenced at the London Research Institute, using the same methodology but on rapid runs.

### Data availability

Exome sequencing data have been deposited in the European Genome-phenome Archive under accession number EGAS00001000907.

### Bioinformatics analysis

We received paired-end reads from the Beijing Genomics Institute in FastQ files, which underwent initial quality control before alignment to the human genome hg19. Coverage levels of aligned files were assessed before and after removal of positional duplicates. Exome capture was performed to an average coverage of 86× and 85× for the right-sided and left-sided tumors, respectively. Somatic single nucleotide variants (SNVs) were detected using the CaVEMan algorithm and germline DNA from blood as the normal reference. A set of *post hoc* filters [9] was then applied to the putative variant calls and the resulting list manually reviewed on the Integrated Genome Viewer [51] to remove variants in poorly aligned reads or those with profiles characteristic of sequencing errors [52]. Sequencing data from normal tissue from both kidneys acted as an additional control against which mutations in tumor tissue were compared; however, no mutations apart from that in *VHL* were identified in normal tissue. Variants were annotated as synonymous or non-synonymous using both dbNSFP [53] and Annovar [54] . Small insertions and deletions were identified using a modified version of Pindel software and methods published previously [9,12,55].

### Validation

We created custom Ampliseq (Lifetech, Paisley, United Kingdom) validation panels by entering genomic positions of all non-synonymous somatic mutations and indels called in at least one region into the Ion AmpliSeq Designer [56] (150 bp amplicon size option selected). Multiplex PCRs were performed according to the manufacturer’s instructions with the tumor-specific primer pool on DNA from each region of the tumors. Amplicon pools were used for the construction of barcoded sequencing libraries and these were multiplex sequenced with 200 bp read length on the Ion Torrent PGM sequencer, to a mean target depth of 500× (Lifetech).

### Ploidy profiling

A suspension of nuclei was created from fresh tumor tissue and washed with phosphate-buffered saline (PBS), then fixed with 70% ethanol. After 60 minutes, nuclei were washed again with PBS and stained with propidium iodide. Flow cytometric analysis of DNA content was performed using the BD LSRFortessa Cell Analyzer, BDFacsDiva™ software and FlowJo software (Oxford, United Kingdom and Ashland, Oregon, United States. The DNA index of the aneuploid peak, where present, was calculated by dividing the G1 peak of the aneuploid population by the G1 peak of the normal diploid cells.

### Copy number analysis

Raw copy number estimates were generated from Illumina exome sequencing data using VarScan2 (v2.2.11) with default parameters except for the data-ratio parameter, which was calculated as in [57]. Low mapability regions (segmental duplications and ENCODE ‘DAC blacklisted’ regions, as annotated by the UCSC genome browser) and the sex chromosomes were excluded. Raw log2-ratio (logR) calls were adjusted for GC content and quantile normalized. Outliers were detected and modified using Median Absolute Deviation Winsorization prior to patient-specific joint segmentation (gamma = 1,000) [58], to identify genomic segments of constant logR.

To calculate absolute (integer) copy numbers from the relative copy number data, tumor purity and ploidy were estimated using ABSOLUTE (v1.0.6) [21]. The analysis incorporated somatic nucleotide variants with variant allele frequencies (VAF) >5% and >50x coverage identified using Caveman that were independently validated and/or unlikely to represent technical artifacts (as determined by varSLR, which models strand bias, mapping quality, base quality and position-in-read in a stepwise logistic regression framework Minimum/maximum ploidy was set to within ±0.5 of the prior ploidy estimate, calculated from the sample’s fluorescence-activated cell sorting (FACS)-based DNA index. To identify an optimal ABSOLUTE model for a given sample, the top five ABSOLUTE models (ranked by log-likelihood) were retrieved. Subsequently, a set of inter-sample models was identified that minimized the total pairwise distance derived from the segments’ expected modal copy number and SNV multiplicity, whilst maximizing the models’ posterior log likelihoods, using rank aggregation (Cross-Entropy method) as implemented in the RankAggreg package [59]. Final model solutions were manually reviewed as recommended (v1.0.6) [21]. Having identified optimal models that fit the relative copy number data, adjacent segments of equal clonality and absolute copy number were merged. LogR plots were annotated with the locations of recurrent copy number changes referenced in the TCGA Copy Number Portal [60]. ABSOLUTE was also used to assess the clonality of SNVs and SCNAs, as specified in [61] (Pr(CCF < 0.95) < 0.5).

To calculate the mirrored B-allele frequency (mBAF), candidate heterozygous SNPs with >40× coverage in the tumor samples were identified in the reference sample using VarScan2, and then filtered by Gaussian mixture model-based clustering using the mclust package [62]. ABSOLUTE-derived purity estimates were used to adjust mBAF, as described by Van Loo *et al*. [63]. In order to identify segments of significant allelic imbalance, the mBAF distributions within a segment between reference and tumor sample were compared using the Wilcoxon signed rank test with subsequent Bonferroni correction.

### mTOR catalytic domain structure modeling

The location of mTOR mutations was mapped using the structure of a large mTOR fragment bound to mLST8 (PDB code 4JSP) and the figure was prepared using the graphics program PYMOL [64].

### Co-transfection assays

We plated 1.8 × 106 HEK-293 T cells per well in six-well tissue culture plates, transfected 24 hours later with Flag-mTOR encoding plasmids (1,500 ng) and HA-S6K encoding plasmid (50 ng) using Lipofectamine 2000 (Life Technologies, New York, New York, United States). Twenty-four hours post-transfection, cells were starved in Hanks Balanced Salt Solution for 1 hour, stimulated in Dulbecco’s modified Eagle medium supplemented with 10% fetal bovine serum for 1 hour, and followed by whole cell lysis in RIPA buffer (50 mM Tris pH 7.5, 150 mM NaCl, 0.1% SDS, 1% Triton X-100, Protease inhibitor cocktail (Roche, New York, New York, United States) and Phosphotase inhibitor cocktail (Millipore, Billerica, Massachusetts, United States).

Whole cell lysates (30 mg) were mixed with NuPAGE LDS Sample Buffer (4X; Life Technologies), heated in 70°C for 10 minutes, loaded into lanes of NuPAGE 4-12% Bis-Tris Protein Gels (Life Technologies), run at 120 volts for 2 hours in 1× MOPS buffer (Life Technologies) and transferred to 0.45 mm PVDF membrane at 100 volts for 1 hour in 1× transfer buffer (25 mM Tris, 192 mM glycine, 20% methanol).

All primary antibodies were diluted 1:1,000 in 1% milk w/v phosphate-buffered saline Tween (PBST), and all secondary antibodies were diluted 1:5,000 in 1% milk w/v PBST. Antibodies were detected using the enhanced Chemiluminescence method (Western Lighting, Perkin Elmer, Waltham, Massachusetts, United States). Immunoblot signals were acquired with the LAS-3000 Imaging system (FujiFilm).

Primary antibodies used were p-p70S6K(Thr389), p70S6K, p-4E-BP1(Thr70), 4E-BP1, Raptor (Cell Signaling, Ozyme, France), Flag-M2 (Sigma, St Louis, Missouri, United States). pRK7-HA-S6K1-wt was a gift from the Neal Rosen lab. pcDNA3-Flag-mTOR wt was acquired from Addgene (Cambridge, Massachusetts, United States).

*MTOR* point mutations were generated in pcDNA3-Flag-mTOR wt using site-directed mutagenesis with the QuikChange II XL kit according to the manufacture’s protocol (Agilent, Santa Clara, California, United States). Primer sequences were: L2427P_F:catcagcctccagttcggcaaggggtcatagac; L2427P_R:gtctatgaccccttgccgaactggaggctgatg; T1652A_F:cttgagccaggctctcatgtcttcatgagggct; T1652A_R: agccctcatgaagacatgagagcctggctcaag.

### Assessment of the phosphokinome

Whole proteins were extracted from each of the four tumors and from two normal kidney samples. The levels of total and phosphorylated forms of intracellular kinases Akt/p-Akt, mTOR/p-mTOR, p70S6K/p-p70S6K and 4E-BP1/p-4E-BP1 were measured using MSD® (Rockville, MD 20850, US) immunoassay technology. Assays were performed by coating 96-well microplates (MSD) with 10 μg/well of whole protein lysates from tumor or cell samples and incubation with the following primary antibodies: Akt, p-Akt (Ser473), p-Akt (Thr308), mTOR, p-mTOR (Ser2448), p70S6K, p-p70S6K (Thr389), 4E-BP1 and p-4E-BP1 (Thr70) (Cell Signaling). Plates were then washed with PBS-T and incubated with SULFO-TAG anti-rabbit or anti-mouse antibodies (MSD) as secondary detection antibody for 1 hour at room temperature. The signal was detected using a SECTOR® Imager 2400 instrument (MSD) and the results expressed in electrochemiluminescence counts. The signal for each sample signal was adjusted for its corresponding GAPDH value. The human serum signal (background) for each of the corresponding antibodies was subtracted. An alternative ratio of phosphorylated to total protein was calculated for each spot, to yield four values for a given sample and a given protein, and then bootstrapping applied to identify the most robust value.

### Immunohistochemistry

Rabbit anti-phospho-S6 ribosomal protein (Ser235/236) and rabbit anti-phospho-4E-BP1 antibodies (Thr37/46) (Cell Signaling: #2211, #2855), were used for immunohistochemistry on paraffin sections. Antigens were unmasked by microwaving in Tris-EDTA pH9 (pS6) and citrate pH6 (4E-BP1) and incubated with primary antibodies at 1:50 (pS6) and 1:800 (p4E- BP), respectively. After incubation in biotinylated secondary antibody and 14Avidin Biotin Complex, slides were developed in DAB substrate (all from Vector, Peterborough, United Kingdom). Staining for pS6 and p4E-BP1 was scored by one pathologist (GS) and grouped into three categories based on staining intensity and frequency [65]: 0, less than 10% of cells with weak staining; 1, more than 10% of cells with weak staining or less than 20% of cells with strong staining; 2, more than 20% of cells with strong staining.

## Additional files

Additional file 1: Table S1. Validated non-synonymous mutations for each tumor. **Table S2.** Validated non-synonymous mutations in tumor in patient 2. **Table S3.** Immunohistochemical analysis of all tumor regions for phosphorylated S6K and 4E-BP1. Tumor staining for each region was assigned a category of 0 (<10% of cells with weak staining), 1 (>10% of cells with weak staining or <20% of cells with strong staining) or 2 (>20% of cells with strong staining). Additional file 2: Figure S1. Copy number profiles for all tumor regions of a young patient with VHL syndrome and multi-focal ccRCC tumors. Additional file 3: Figure S2. Ploidy profiles for four distinct tumors, confirming genome doubling in tumor four with a DNA index of 1.82. Additional file 4: Figure S3. Clinical photographs from partial nephrectomy for a stage 1 ccRCC in a 67-year-old patient with VHL syndrome. The tumor was located in the lower anterior pole of the right kidney and was bisected to give two tumor regions. Additional file 5: Figure S4. Heatmap showing validated non-synonymous mutations in a morphologically homogeneous tumor from a 67-year-old patient with VHL syndrome. The presence (blue) or absence (grey) of each mutation is indicated for tumor regions 1 and 2. Additional file 6: Figure S5. Kinase mutant induces S6K1 phosphorylation. HEK-293 T cells were co-transfected with HA-S6K and mTOR wt or mutant cDNA plasmids. Twenty-four hours later, cells were nutrient-deprived for 1 hour, nutrient-stimulated for 1 hour, followed by whole cell lysis. The lysates were then immunoblotted for the indicated proteins and phosphorylation states of S6K1 and 4E-BP1. Additional file 7: Figure S6. Representative slides demonstrating positive staining for S6K and 4E-BP1 in tumors 2 and 3. **(A)** S6K staining of tumor two at 10× magnification, **(B)** 4E-BP1 staining of tumor two at 40× magnification, **(C)** S6K staining of tumor three at 10× magnification, and **(D)** 4E-BP1 staining of tumor three at 10× magnification. Additional file 8: Figure S7. Mutual exclusivity analysis showing a strong tendency towards mutual exclusivity of mutations in *ARID1A*, *MTOR*, *TSC1* and *TSC2.* Each grey bar represents one tumor biopsy reported by the TCGA (image cropped and magnified; dataset analysed included 417 tumor samples). Mutations in *MTOR*, PIK3CA, ARID1A, PTEN, TSC1 and TSC2 are indicated by green bars. Figure generated using cBio cancer genomics portal.

## Abbreviations

bp: base pair
ccRCC: clear cell renal cell carcinoma
CNA: copy number aberration
FAT: FRAP-ATM-TRAAP
ITB: index of branch to truncal mutations
ITH: intra-tumor heterogeneity
LOH: loss of heterozygosity
mBAF: mirrored B-allele frequency
MSD: Meso-Scale Discovery
M-seq: multi-region sequencing
mTOR: mammalian target of rapamycin
PBS: phosphate-buffered saline
SNV: single nucleotide variant
TCGA: The Cancer Genome Atlas
VHL: von Hippel-Lindau
WES: whole exome sequencing
