# Supplementary material for: Development of synchronous VHL syndrome tumors reveals contingencies and constraints to tumor evolution
Source: Genome Biol. 2014 Aug 27;15(8):433. doi: 10.1186/s13059-014-0433-z (PMC4166471; doi:10.1186/s13059-014-0433-z)
Supplement: Additional file 1: Table S1. — Validated non-synonymous mutations for each tumor. Table S2. Validated non-synonymous mutations in tumor in patient 2. Table S3. Immunohistochemical analysis of all tumor regions for phosphorylated S6K and 4E-BP1. Tumor staining for each region was assigned a category of 0 (<10% of cells with weak staining), 1 (>10% of cells with weak staining or <20% of cells with strong staining) or 2 (>20% of cells with strong staining). [file 13059_2014_433_MOESM1_ESM.docx]

| **Gene** | **Chromosome** | **Position** | **Nucleotide** | **Amino acid change** |
| --- | --- | --- | --- | --- |
| **TUMOUR 1** |  |  |  |  |
|  |  |  |  |  |
| ARID1A | 1 | 27099857 | A>G | M1246V |
| VPS13B | 8 | 100836181 | T>A | V3127D |
| NFKBIE | 6 | 44232861 | A>T | S214T |
| KIAA0240 | 6 | 42832616 | G>C | G891A |
| GPLD1 | 6 | 24429318 | Del:GAG | R821 RS>S |
| COL25A1 | 4 | 110222994 | G>T | A61E |
| DDX49 | 19 | 19031434 | C>A | A54E |
| NANOS3 | 19 | 13988241 | C>A | P60H |
| C17orf53 | 17 | 42230009 | Ins:T | V438fs*3 |
| GRIN2A | 16 | 9923410 | G>A | T626I |
| MPHOSPH8 | 13 | 20224398 | C>T | S525L |
| RFX4 | 12 | 107103125 | G>T | R293L |
| PZP | 12 | 9309894 | C>T | E1143K |
| TRAPPC4 | 11 | 118889998 | G>T | E107D |
| C1orf131 | 1 | 231374872 | C>A | D61Y |
| LINGO4 | 1 | 151774588 | A>G | L198P |
|  |  |  |  |  |
| **TUMOUR 2** |  |  |  |  |
|  |  |  |  |  |
| RAB11FIP1 | 8 | 37734931 | Del:T | F170fs*4 |
| PIK3C2A | 11 | 17135955 | G>A | R1092C |
| LAMC3 | 9 | 133966975 | G>T | W1510L |
| CEP78 | 9 | 80861644 | C>A | L280I |
| ASCC2 | 22 | 30198112 | G>T | P404Q |
| ZNF217 | 20 | 52199037 | T>A | K110I |
| KLF11 | 2 | 10192452 | A>T | K436X |
| TMEM38A | 19 | 16797133 | T>G | F197V |
| HOXB5 | 17 | 46670995 | G>T | P17Q |
| RTN4RL1 | 17 | 1839996 | C>A | A374S |
| IDH2 | 15 | 90634844 | C>T | V50M |
| BRF1 | 14 | 105677503 | DEL:GGA | E446 ED>D |
| ARHGAP23 | 17 | 36623053 | C>T | R377W |
| LRRC27 | 10 | 134165214 | C>T | R344X |
| UTP20 | 12 | 101700487 | Del:TGACAA | L648 LTI>L |
| GRIK4 | 11 | 120702637 | C>A | D196E |
| ANXA11 | 10 | 81923154 | C>T | R346H |
|  |  |  |  |  |
| **TUMOUR 3** |  |  |  |  |
|  |  |  |  |  |
| LPPR1 | 9 | 104048501 | Del:G | R123fs*3 |
| EDEM1 | 3 | 5229617 | T>A | F43I |
| TOP1 | 20 | 39690034 | A>C | D20A |
| MTOR | 1 | 11174395 | A>G | L2427P |
| TK1 | 17 | 76181237 | C>A | L36F |
| CCDC77 | 12 | 542377 | A>G | K205R |
| CATSPER1 | 11 | 65790407 | C>G | A448P |
| EPC1 | 10 | 32594789 | G>C | P75R |
| PLXDC2 | 10 | 20106053 | G>C | M15I |
| CUBN | 10 | 17147472 | A>C | L405R |
| PBRM1 | 3 | 52663054 | G>C | Splice |
| NRAS | 1 | 115252341 | A>G | I100T |
| KDM6A | X | 44941968 | G>A | R1073K |
|  |  |  |  |  |
| **TUMOUR 4** |  |  |  |  |
|  |  |  |  |  |
| SPATC1 | 8 | 145095085 | G>A | G163S |
| ABCA13 | 7 | 48311958 | A>G | I899V |
| MYB | 6 | 135518253 | A>T | K453I |
| USP45 | 6 | 99893724 | C>T | E642K |
| CRISP3 | 6 | 49705042 | T>C | D36G |
| ATP10B | 5 | 160044969 | T>C | N810D |
| ANXA3 | 4 | 79507442 | T>C | I114T |
| L3MBTL2 | 22 | 41621916 | A>T | K492M |
| STAT1 | 2 | 191848465 | G>A | T450M |
| SLC25A12 | 2 | 172666195 | A>G | V409A |
| TBXA2R | 19 | 3600457 | G>A | T59M |
| PTBP1 | 19 | 810617 | T>A | F506Y |
| PRTG | 15 | 55933360 | T>C | I696M |
| EXD1 | 15 | 41476565 | G>T | S370Y |
| SLC39A9 | 14 | 69922535 | INS:A | A215fs*17 |
| MCF2L | 13 | 113742709 | Del:G | Q919fs*47 |
| NALCN | 13 | 101710346 | A>T | D1656E |
| MTOR | 1 | 11199634 | T>C | T1652A |
| MEGF6 | 1 | 3422046 | C>A | D665Y |
| TAOK3 | 12 | 118671521 | T>A | K201X |
| KCNA6 | 12 | 4919649 | G>T | E148X |
| OR51L1 | 11 | 5020637 | T>C | V142A |
| NHLH2 | 1 | 116380938 | G>C | S19W |

**Supplementary table 1:** Validated non-synonymous mutations for each tumour

| **Gene** | **Chromosome** | **Position** | **Nucleotide** | **Amino acid change** |
| --- | --- | --- | --- | --- |
| SPIN4 | X | 62569995 | T>A | D235V |
| OPN3 | 1 | 241761216 | T>A | L259F |
| RPE | 2 | 210880966 | T>G | S59R |
| GHDC | 17 | 40344368 | C>G | Q260H |
| TSC2 | 16 | 2129071 | C>T | S1002F |
| HSPA14 | 10 | 14894507 | G>T | Q237H |
| GPLD1 | 6 | 24450049 | G>A | P472S |
| MARCH8 | 10 | 45953911 | G>A | Q218X |
| USP54 | 10 | 75265178 | G>T | A1353E |
| SYT3 | 19 | 51133064 | G>C | R347G |
| PPRC1 | 10 | 103899014 | A>G | E250G |
| HEATR5B | 2 | 37276944 | G>T | R850S |
| WNK1 | 12 | 1017953 | A>C | T2642P |
| CRAT | 9 | 131862150 | C>A | E339D |
| MAD2L1BP | 6 | 43608095 | G>A | G249E |
| PROSER1 | 13 | 39587933 | A>G | S464P |
| CERS2 | 1 | 150938640 | T>C | H376R |
| NCKAP5 | 2 | 133540306 | T>C | S1360G |
| NCOA1 | 2 | 24914366 | G>A | W183X |
| ADAM15 | 1 | 155030554 | G>T | Q548H |
| TNR | 1 | 175304883 | C>T | V1199I |
| PIPOX | 17 | 27383315 | C>A | H389N |
| AHDC1 | 1 | 27875745 | T>G | H961P |
| LCA5L | 21 | 40777815 | A>T | I669N |
| EML6 | 2 | 55098719 | G>T | G774V |
| PHF15 | 5 | 133873705 | A>T | R29X |

**Supplementary table 2:** Validated non-synonymous mutations in tumour in patient 2

| Tumour | | Region | **pS6K** | **P4E-BP1** |
| --- | --- | --- | --- | --- |
| Right kidney | One | R1 | 2 | 0 |
|  |  | R8 | 1 | 0 |
|  | Two | R2 | 1 | 0 |
|  |  | R3 | 1 | 0 |
|  |  | R4 | 1 | 0 |
|  |  | R6 | 2 | 1 |
|  |  | R7 | 1 | 0 |
|  | Normal | N | 0 | 0 |
| Left kidney | Three | R1 | 1 | 2 |
|  |  | R2 | 1 | 2 |
|  |  | R3 | 1 | 2 |
|  |  | R4 | 2 | 2 |
|  |  | R5 | 2 | 2 |
|  |  | R6 | 2 | 2 |
|  |  | R7 | 1 | 2 |
|  |  | R8 | 2 | 2 |
|  | Four | R9 | 2 | 2 |
|  | Normal | N | 0 | 0 |

**Supplementary table 3:** Immunohistochemical analysis of all tumour regions for phosphorylated S6K and 4E-BP1. Tumour staining for each region was assigned a category of 0 (< 10% of cells with weak staining), 1 (> 10% of cells with weak staining or < 20% of cells with strong staining) or 2 (> 20% of cells with strong staining).
