# Supplementary figures and images for: Development of synchronous VHL syndrome tumors reveals contingencies and constraints to tumor evolution
Source: Genome Biol. 2014 Aug 27;15(8):433. doi: 10.1186/s13059-014-0433-z (PMC4166471; doi:10.1186/s13059-014-0433-z)

RK24

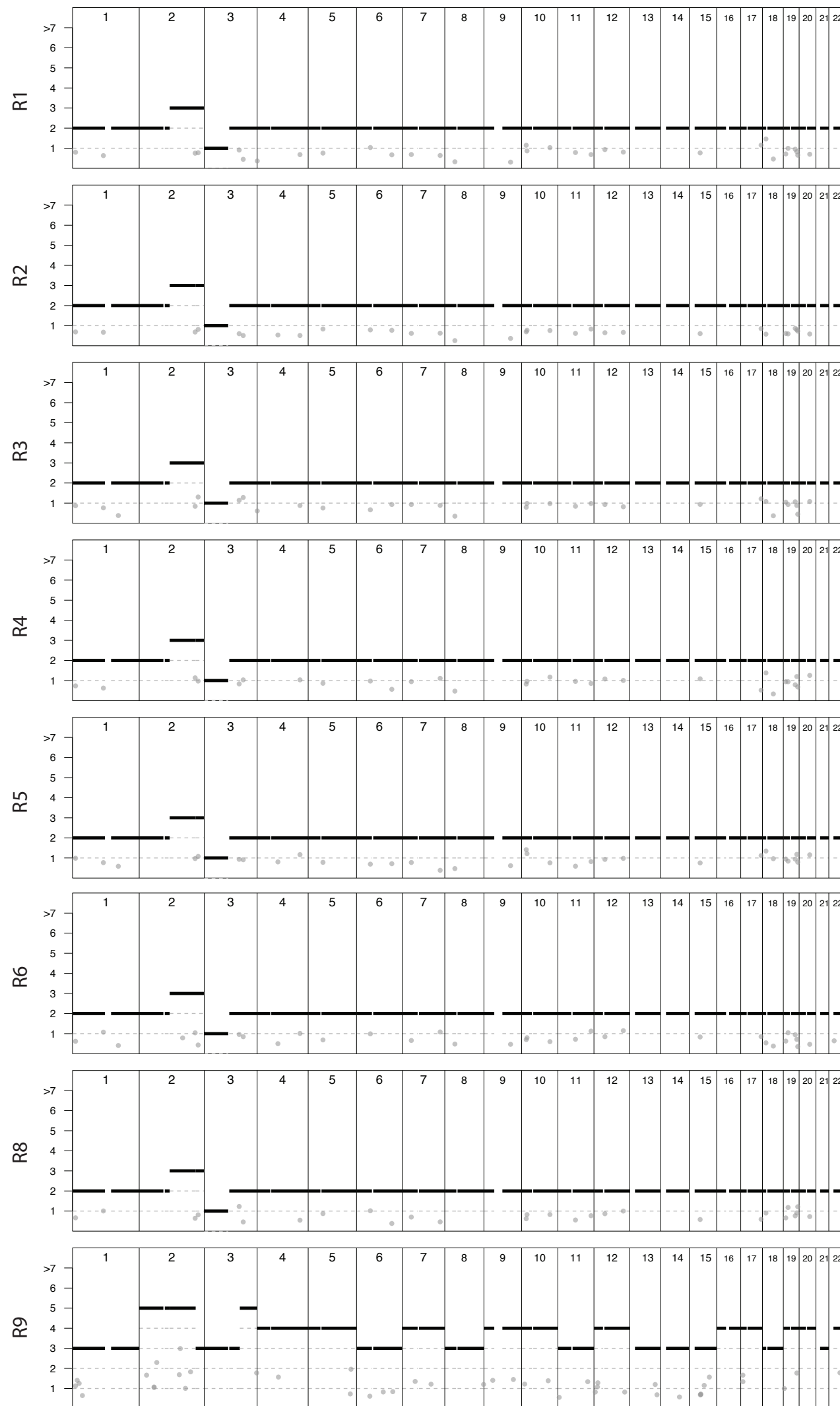

CK24

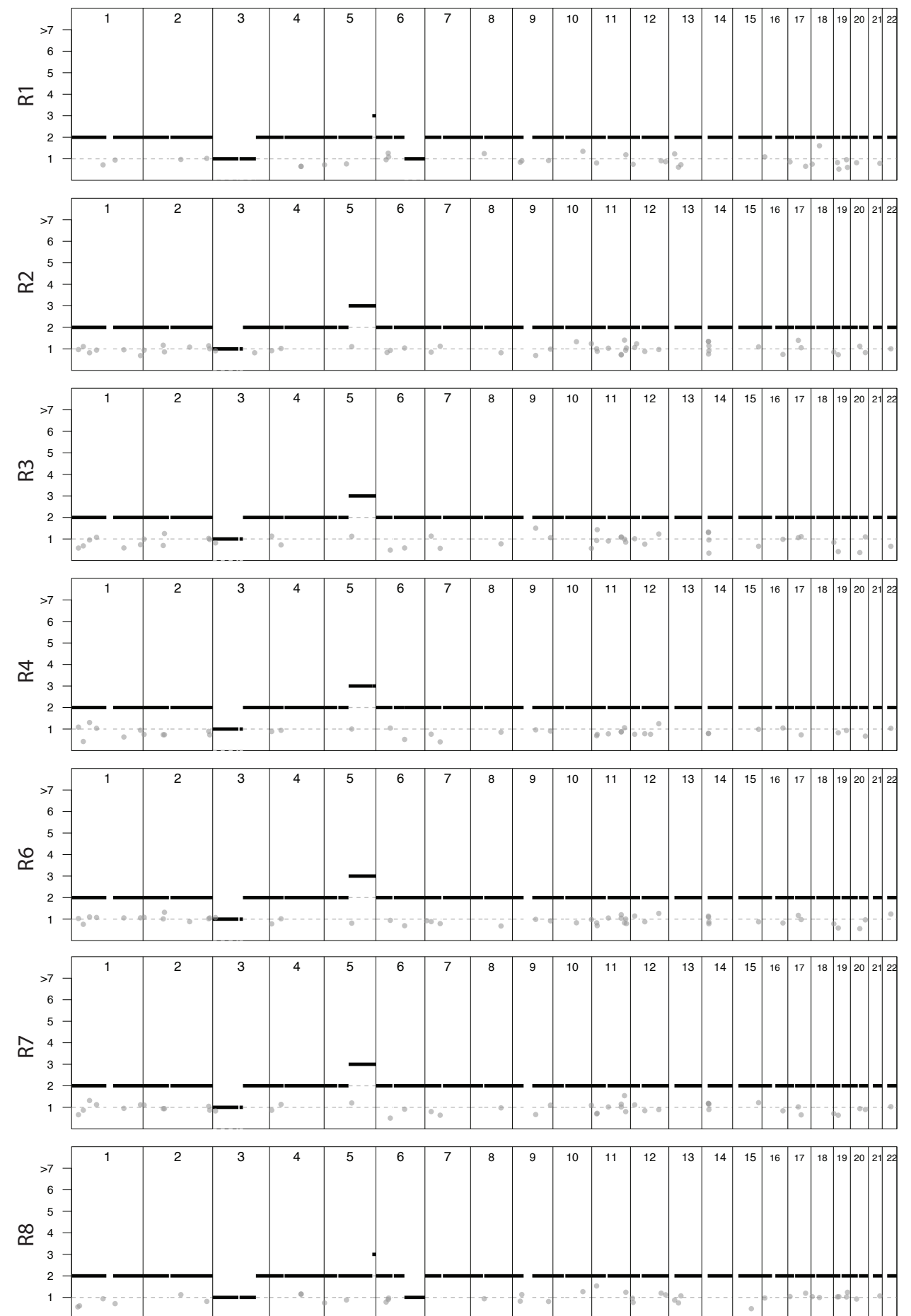

Supplement: Additional file 2: Figure S1. — Copy number profiles for all tumor regions of a young patient with VHL syndrome and multi-focal ccRCC tumors. [file 13059_2014_433_MOESM2_ESM.pdf]

## right kidney

## left kidney

### Tumour 1

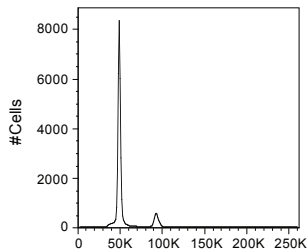

### Tumour 3

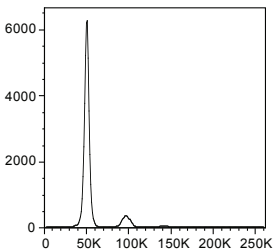

### Tumour 2

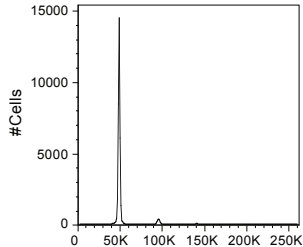

### Tumour 4

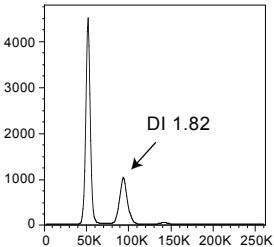

### Normal

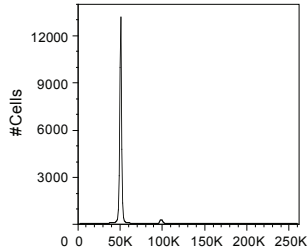

### Normal

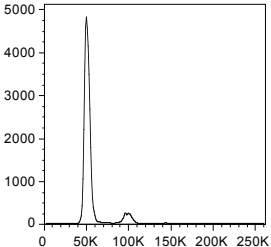

Supplement: Additional file 3: Figure S2. — Ploidy profiles for four distinct tumors, confirming genome doubling in tumor four with a DNA index of 1.82. [file 13059_2014_433_MOESM3_ESM.pdf]

A)

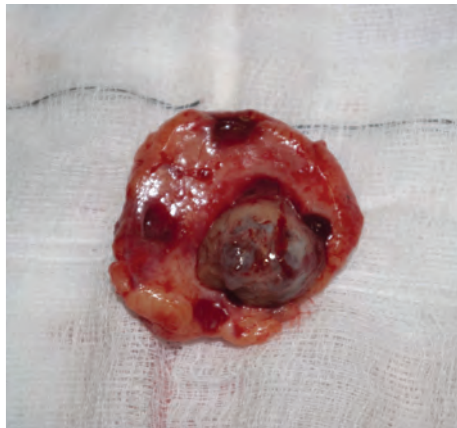

B)

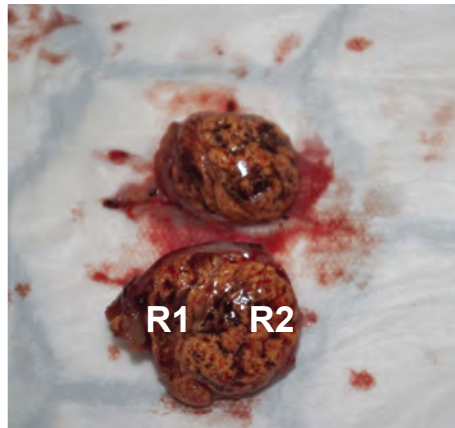

Supplement: Additional file 4: Figure S3. — Clinical photographs from partial nephrectomy for a stage 1 ccRCC in a 67-year-old patient with VHL syndrome. The tumor was located in the lower anterior pole of the right kidney and was bisected to give two tumor regions. [file 13059_2014_433_MOESM4_ESM.pdf]

A

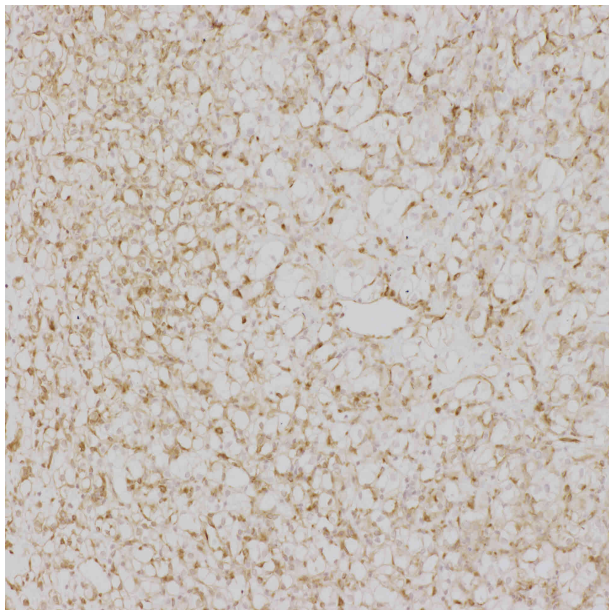

B

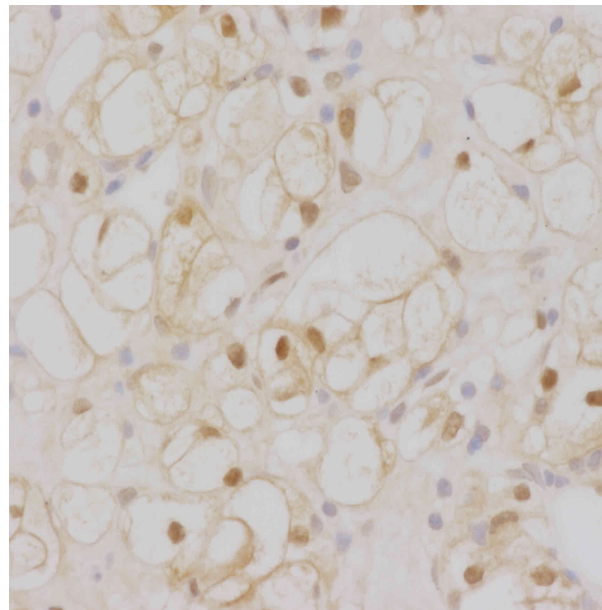

C

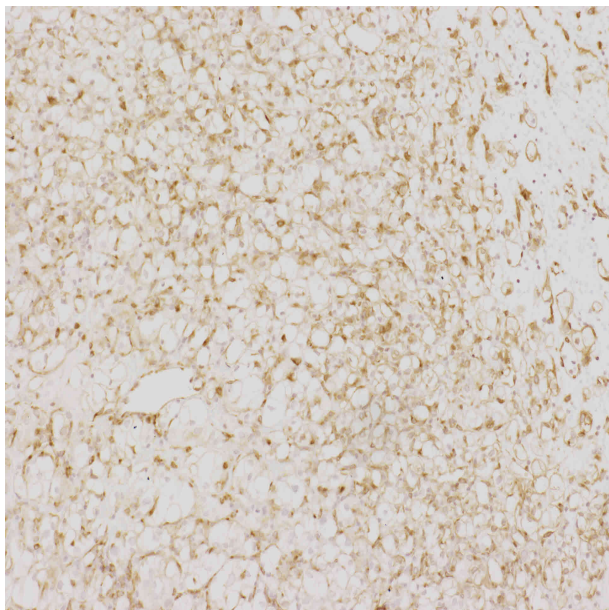

D

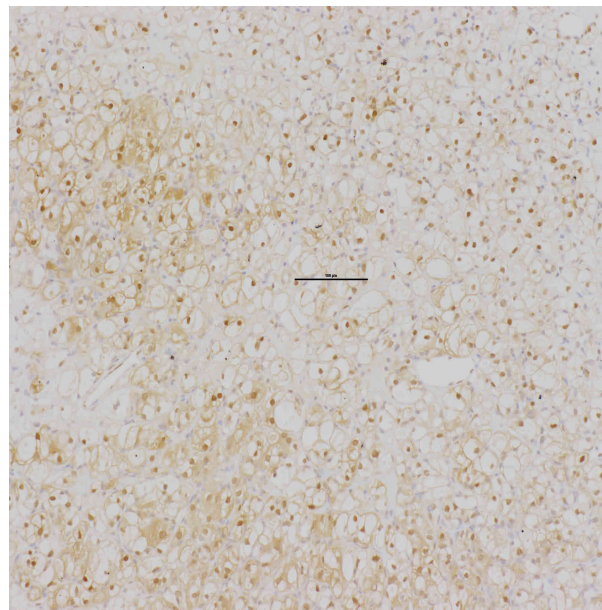

Supplement: Additional file 7: Figure S6. — Representative slides demonstrating positive staining for S6K and 4E-BP1 in tumors 2 and 3. (A) S6K staining of tumor two at 10× magnification, (B) 4E-BP1 staining of tumor two at 40× magnification, (C) S6K staining of tumor three at 10× magnification, and (D) 4E-BP1 staining of tumor three at 10× magnification. [file 13059_2014_433_MOESM7_ESM.pdf]

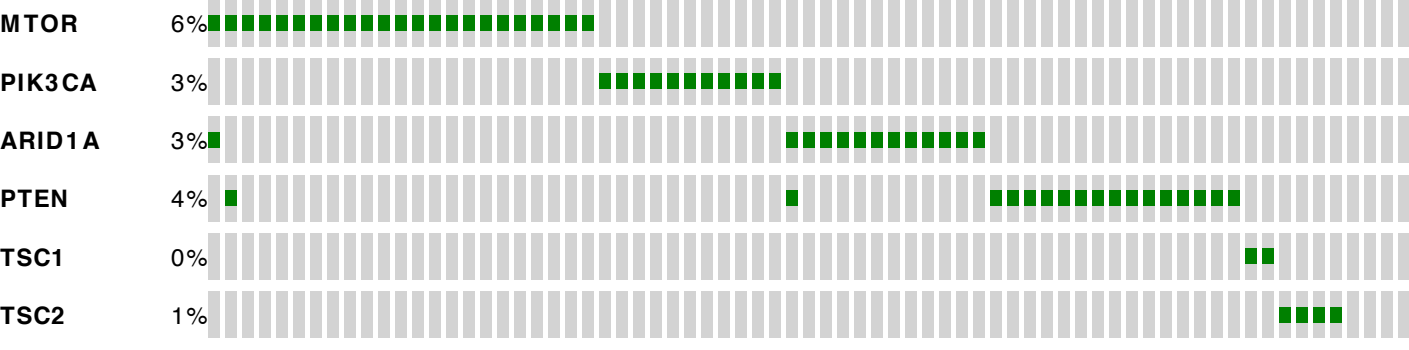

Supplement: Additional file 8: Figure S7. — Mutual exclusivity analysis showing a strong tendency towards mutual exclusivity of mutations in ARID1A, MTOR, TSC1 and TSC2. Each grey bar represents one tumor biopsy reported by the TCGA (image cropped and magnified; dataset analysed included 417 tumor samples). Mutations in MTOR, PIK3CA, ARID1A, PTEN, TSC1 and TSC2 are indicated by green bars. Figure generated using cBio cancer genomics portal. [file 13059_2014_433_MOESM8_ESM.pdf]
