# Supplementary material for: Development of synchronous VHL syndrome tumors reveals contingencies and constraints to tumor evolution
Source: Genome Biol. 2014 Aug 27;15(8):433. doi: 10.1186/s13059-014-0433-z (PMC4166471; doi:10.1186/s13059-014-0433-z)
Supplement: Additional file 5: Figure S4. — Heatmap showing validated non-synonymous mutations in a morphologically homogeneous tumor from a 67-year-old patient with VHL syndrome. The presence (blue) or absence (grey) of each mutation is indicated for tumor regions 1 and 2. [file 13059_2014_433_MOESM5_ESM.pdf]

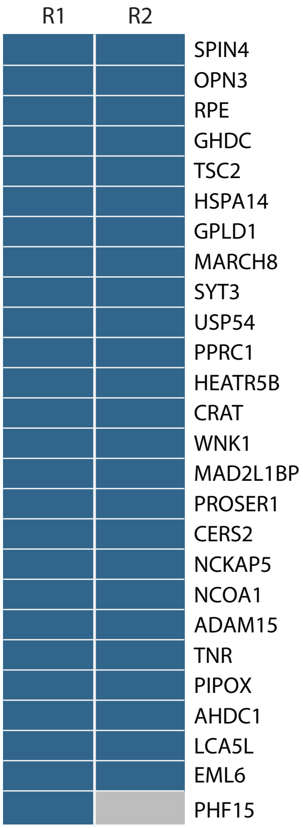
R1
R2
SPIN4
OPN3
RPE
GHDC
TSC2
HSPA14
GPLD1
MARCH8
SYT3
USP54
PPRC1
HEATR5B
CRAT
WNK1
MAD2L1BP
PROSER1
CERS2
NCKAP5
NCOA1
ADAM15
TNR
PIPOX
AHDC1
LCA5L
EML6
PHF15
